# Selections and Abstracts

**Published:** 1917-02

**Authors:** 


### SELECTIONS AND ABSTRACTS

### SOME OF THE LESSONS LEARNED BY THE RECENT MOBILIZATION ON THE BORDER.

The necessity for the rapid mobilization of the National Guard regiments and their muster into the Federal service, before leaving their State camps, placed an unexpected burden upon the medical militia officer.

While the writer has no definite knowledge regarding the sequence of events in the mobilization camps of other states, it is safe to assume that the conditions which obtained in Vermont were not dissimilar to those which prevailed elsewhere.

The medical militia officers had orders from the War Department to give each officer and enlisted man a careful physical examination before mustering him into the Federal Service and to proceed with these examinations without delay. This we did, but to do so promptly we were forced to strip our private offices of scales, measuring-rods, Snellen's test-cards and other paraphernalia necessary for such careful examinations, as no such equipment was provided at the mobilization camps.

In justice to the Medical Supply Depot at Washington, I can testify that within eight days after our requisitions were made, and in spite of the fact that it was swamped with telegrams from every state in the Union, a field hospital, two camp infirmaries and ample quantities of protective vaccines were delivered to us, and this promptness continued without a break during the entire summer.

The tenth day after the call came found the Vermont regiment entrained for the Border and I believe it was the first New England regiment to arrive there.

Our six medical officers examined approximately 1,200 men, of whom about 10 per cent. were rejected. As these examinations went on day and night many guardsmen were passed who should have been disqualified, but a tent full of noisy men and a smoky lantern held by an untrained boy were not conditions conducive to accurate diagnosis· Had we adhered strictly to the regulations laid down by the War Department a still greater percentage would have been rejected, but for one of several reasons many were accepted and their physical defects noted upon their examination blanks.

The potency of the small-pox vaccine was a revelation and many men who deemed themselves protected "took" with amazing promptness. About 10 per cent. of the men were acutely ill for three or four days and 40 per cent. of them nursed a sore arm for as many weeks.

Out of approximately 3,500 hypodermic injections of typhoid vaccine no abscess resulted· They were, when possible, given about 4 p. m., and the soldier excused from duty next day. No serious morbidity resulted from these protective injections, although 20 per cent. of the men complained of headache and muscular soreness the day following the exhibition of the vaccine. The refinements of this method of protection make small-pox vaccination seem crude and barbaric in comparison.

The following were the causes of rejection: Acute illness, 1; appendicitis, 2; asthma, 1; burns and other scars, 2; cardiovascular system, 13; defective vision, 10; defective teeth, 8; chancre, 1; diseases of the skin, 1; enlarged glands, 1; flat feet, 2; gonorrhea, 3; hernia, 8; hemorrhoids, 1; phimosis, 1; stiff leg, 1; tuberculosis, 2; simple urethritis, 3; undersize, 5; varicocele, 9; and about 40 recruits whose names were not carried upon the company's muster roll and of whom no record was kept were rejected.

Without going into the causes of these disabilities in detail it might be of interest to say that with few exceptions hernias and varicoceles were on the left side. This fact had not been brought to the writer's notice and a perusal of medical and surgical text-books failed to reveal any reference to the cause of such left-sided conditions. A possible explanation might be that varicocele is a thrombotic condition of the vessels of the pampiniform plexus which unite at the internal inquinal ring to form the spermatic vein. These veins are poorly supplied with valves, and while the right spermatic vein empties directly into the inferior vena cava, the left empties into the left renal vein. Add to this indirect circulation the fact that the left spermatic cord is longer, and as a consequence the left testicle hangs lower than the right, and we might with reason put forth this theory as a cause of the prevalence of left-sided varicocele, and the pressure atrophy these enlarged veins exert upon the left inguinal internal ring and upon the inguinal canal might be a causative factor of left-sided hernias.

It is not my desire to compare the relative value to the United States government of the regular and the militiaman, and the discussion of the merits of volunteer and compulsory enlistments should be left to minds attuned to matters military, but I would crave the privilege of submitting proofs that the national guardsman on the Border was physically fietter than his brother, the regular.

No argument is needed to show that the regular sodier has the advantage of the militiaman in drill and discipline, but all must admit that in an emergency the latter would be superior to the former on account of higher mentality and initiative. From a perusal of recent army reports (which by the way have not lacked publicity) the idea, however, has become crystallized in the mind of the casual reader that the militiaman on the Border was a hopeless cripple, whose favorite habitat was the hospital, and had the invasion of Mexico taken place would have "destroyed himself" (I believe this is the favorite phrase) on schedule time.

History, nevertheless, records that the President's call found the National Guard ready, but not equipped, and in a very few days the men were on their way to the Mexican border.

The militia medical officer is criticized in no uncertain terms for allowing many of these guardsmen to qualify. One of these reports is before me as I write. It goes on to say that we sent the lame, the halt and the blind, the senile and the immature, and in one case a guardsman was found who wore an artificial leg.

Let us consider for a moment how the products of our very cursory examination stood the gaff during the summer. When the National Guard arrived on the Border, the men did the same work and took the same hikes as the regulars, in spite of the fact that many of them had their prophylaxis against small-pox and typhoid on the train or shortly after their arrival. Take into consideration that they were all raw, unseasoned troops, that they were continually insulting their stomachs with impure food bought to supplement the government ration to which they had been unaccustomed, that many of them had left families who were soon to be in want, and for several other reasons inimical to their health and happiness, and you will get a faint idea of what the National Guard was up against. Remember, they were encamped on the Devil's playground—where every tree had thorns, where croaking toads replaced the carol of the birds, where flies and fleas and mosquitoes annoyed, where the snake, the scorpion and the tarantula gambolled over them as they slept, where sun and sand and wind and rain buffeted—where—but their country called and they responded.

In round numbers there were stationed from Yuma to Brownsville 110,000 militia and 40,000 regulars. Out of this number, from July 1st to October 1st, there were discharged for disability 151 regulars and 878 militia—0.38 per cent. for the regulars and 0.79 per cent. for the guardsmen. This on its face would show that the relative percentage of guardsmen discharged for disability was over double that of the regulars, but we must take into consideration that many of the former were dismissed under the heading, "disability," the real reason being a business or personal one.

On August 30, 1916, there were sick in hospital 832 regulars (20 to 1,000) and 1,284 militia (11 to 1,000). On September 30, 1916, there were sick in hospital 950 regulars (21 to 1,000) and 1,585 guardsmen (14 to 1,000). On October 30, 1916, there were sick in hospital 1,016 regulars (25 to 1,000) and 1,571 guardsmen (14 to 1,000). During the month of October, 1916, there were stationed on the Border 39,543 regulars and 102,150 militia. The death-rate for this month was 12 for the regulars and 17 for the militia. The fact that six of these deaths among the militia resulted from accidental shot-wounds, three from injury, and one from drowning, would show that the mortality from actual disease among the National Guard was about one-third that of the regulars. These statistics are authentic and form a portion of the report of the Assistant Surgeon to the Southern Department printed in the "Journal of the American Medical Association."

The regulars were as a rule $15.00 a month men, many of whom had failed to secure employment elsewhere, while 15 per cent. of the guard were business or professional men, 50 per cent. more were artisans and farmers, and the remainder were from the stratum of society equal at least to that of the regular. Thus it is not strange that the work of policing was less arduous among the militia. In plain words, the number under arrest from July 1, 1916, until November 1, 1916, was, relatively speaking, greater among the regulars than among the National Guard, and many of these arrests among the militia could be attributed to the obstinacy of youth, which a few months of intensive military training would have remedied.

The medical officers of the National Guard are ready to concede that many misfit and weaklings were inadvertently admitted to the federalized militia, but that the guardsman made a good showing during his summer on the Border the foregoing morbidity and mortality records will prove, and if an invasion of Mexico had been deemed necessary, we feel certain that he would not have been found wanting.—Edmond J. Melville, Major., in "International Journal of Surgery.

### A PLEA FOR THE EARLIER RECOGNITION OF SQUINT IN CHILDREN BY THE FAMILY PHYSCIAN, AND THE EARLIER APPLICATION OF THE METHODS OF TREATMENT.

By C. A. Veasey, M. D.
Spokane, Wash.

At a meeting of the Pennsylvania State Medical Society just sixteen years ago, the speaker read a paper with the above title. It would seem that, with the advancement of medical education, including more preparatory work and higher educational standards before one can enter upon the study of medicine in a first-class medical school and, furthermore, from the greater attention that has been paid to detail work in special lines by the medical schools during the past fifteen years, it would be entirely unnecessary to present such a paper at the present time. In my own experience, however, I regret to say that so frequently I meet with cases of squint in children of advanced age that have received no treatment whatever, I have concluded once more to make a plea for the earlier recognition of the affection and the earlier application of the methods of treatment.

It is a well known fact that a large proportion of the cases of squint met with in ophthalmic practice begin in childhood. The period of most frequent occurrence is that age when the little one begins to make accomodative effort, as in looking at picture s, picking up blocks, etc., usually at about the age of three or four years. Some cases are, of course, congenital; others are acquired within the first few weeks of life; others still are paralytic and may appear at any time but the greater number become manifest about the time mentioned.

The family physician, as a rule, has his attention directed to the condition shortly after its appearance. If he is indifferent and says, as unfortunately so frequent is done, "Oh, the child will outgrow it, let it alone," in most cases the squint will become permanent, and a portion of the visual acuity of one eye may be lost. On the contrary, if the proper treatment be instituted, much can be done toward relieving the deformity and ;at the same time preserving the useful vision of both eyes, as well as in avoiding the many neutortic ailments to which eye-strain frequently gives rise.

It is not within the province of this paper to review the different causes of squint. It might perhaps be well to add, however, that it is not caused by looking at a lock of hair falling to one side of the face, by fright, by imitation of another crosse-eyed person, or by "eating a dish of cold beans purloined from the kitchen pantry," all of which have been given me as causes by different parents who have brought me their children in consultation.

The larger number of cases, as previously intimated, depends upon some disturbance between the relation of accommodation and convergence, a condition generally brought about by some error of refraction. It would seem, therfore, that the proper procedure in all cases of squint, except, perhaps, those of paralytic origin, would be to determine whether there is present any error of refraction· If one is familiar with the use of the ophthalmoscope, this can be done readily· If not, one drop of solution of atropin, four grains to the ounce, should be instilled into each eye night and morning, and the child's eyes protected from strong light; in fact, dark glasses should be worn out of doors, if the child is old enough to keep them on. Should the squint disappear or become very much better, it will prove that an error of refraction is present in the case, the strain upon the accomodation having been relieved by the action of the atropin upon the cilary muscle, and the patient should be carefully glased. Should the patient be too young to wear glasses, the atropin instillation in half strength may be kept up at varying intervals in the meantime, in the good eye, in order to make the patient employ the squinting eye. As you perhaps recall, the vision of the squinting eye usually becomes less and less and, according to some authorities, this is because the eye is not used, the bulk of the work being done by the non-squinting eye. If, however, the accommodation of the non-squinting eye be paralyzed, the child must use his squinting eye in order to see well, and this use of the eye assists in preventing the loss of vision which ordinarly occurs in maltended cases of squint.

The youngest patient for whom glasses were prescribed by the writer was two years of age; but frequently he has glassed cases of two and a half and three years and he is quite sure that he has relieved, not only the spuint, but saved the vision in many cases. As a rule, children from three and a half to five years of age wear glasses with very little difficulty, if they have convergent squint.

If the wearing of glasses for several weeks fails to correct the defect or portion of it, a system of muscle gymnastics, known as "orthoptic exercises," should be employed. The apparatus required is an ordinary box sterescope with a series of double pictures. The patient is first taught to recognize both pictures on the cards or to see double, thus showing that both eyes are used at the same time and, after this has been accomplished, to fuse the two pictures into one. This method is of very little use in cases of high degrees of squint in which the glasses produce but little change, but in cases of low degree, or in cases much benefited by the wearing of glasses, or in cases of residual squint after operation, it is decidedly of the greatest value.

Should the wearing of the proper glasses and the faithful systematic employment of orthoptic exercises fail to cure the squint, recourse must be had to operative measures. This usually consists of a tenotomy of one muscle, or an advancement of its opponent or both or some of the various substitutes, depending upon the amount of deviation to be dealt with, the variety of the squint, the strength of the opposing muscle and the condition of the visual acuity of each eye. If the defect is convergent, the eye should not be entirly straightened at the operation but a small amount of residual squint should be permitted to remain, as there is always a slight after-tendency to divergence. It is the writer's custom, therefore, to leave from three to five degrees of convergence in all operations for convergent strabismus. For the same reason, in operating for divergent squint, it is better to produce a very slight overcorrection.

As to the age of the patient, it is ordinarily best to wait until the child is about six years old, though there are a few cases of exceedingly high degrees of squint in which earlier operation is not only advisable but, being performed, the eyes are placed in much better position for the retention of normal vision.

These few brief notes give but a meager outline as to the methods to be followed in the treatment of concomitant convergent strabismus and are offered principally to direct the attention of the family physician to the condition upon its first appearance, so that it will not be put aside as something to be outgrown, but will receive the prompt attention it deserves— Northwest Medicine.

---

### THE TECHNIQUE AND MANAGEMENT OF OPERATIONS ON THE STOMACH

Crile (Annals of Surgery, September, 1916) in the course of remarks on this subject particularly on the circumstance that life is possible only in an alkaline medium and urges that efforts be made to increase the reserve alkalinity, and to diminish or control the production of acids in the operation itself.

The reserve alkalinity may be increased by administering approximately 2000 Cc. of normal saline solution subcutaneously the afternoon and evening before and on the morning of the operation; and giving approximately 100 grains of sodium bicarbonate by mouth or by rectum.

He regards as the most important preoperative procedure a night of natural or artifical sleep. Postoperatively he advises normal saline solution subcutaneously until food is taken and a 5-per-cent solution each of sodium bicarbonate and glucose by the Murphy drip; early propping in bed, early rising from bed, and early return to home. In doubtful cases gastrojejuntostomy should precede resection of the stomach. The loop of jejunum should be long enough to lie easily and comfortably. In the large majortiy of cases the traction of the jejunum to the left or right, or the position of the stoma in the stomach, whether vertical or longitudinal, can have no influence whatsoever upon the passage of the food in the stomach or on the propulsion of the food after it has reached the intestine. The opening in the mesocolon is best made by sharp knife dissection rather than by a blunt perforation or by a tear made by traction. The messocolon opening is sutured an inch up on the stomach wall, so that this portion of the stomach goes down through the mesocolon like a hopper and the freedom of the ends of the jejunum is unhampered. This is preferable to suturing the mesocolon opening upon the line of suture at the stoma between the stomach and the intestines, as in this position it may produce kinking at the point of junction between the ileum and the stomach.

The first line suturing of silk or linen should be avoided. Chromic gut is used for the sutures that come in contact with the mucosa. For the peritoneal layer he uses interrupted silk sutures made as Cushing right-angled stitches. At the point where the stomach and jejunum are approximated two or three carefully placed interrupted sutures are inserted, so that the duodenum is brought in contact with the stomach wall on each side of the incision for a distance of from a half to one inch. The main suture which controls absolutely both hemorrhage and leakage is made with the shoemaker stitch, the introduction of which is facilitated greatly by the Bartlett needle and suture. The shoemaker stitch includes the entire structure and strength of the wall of the stomach and intestine.

In most cases it will be found that the gastrojejunostomy and the interval of adjustment have accomplished for these patients what the delay of a week accomplishes for fractures of the patella; what iodoform gauze packing accomplishes in the first stage of a laryngectomy; what acute salpingitis does for later salpingectomy. In each instance the protective reserves against infection are called out into the local field, and adequate local defense against infection in the later operation is thus guaranteed. Usually very few fragile adhesions will be found, and the entire operative field will be easily mobilized. The gastrojejunostomy will appear as if it were a natural opening. In most instances no pull or strain will be necessary to develop the field, and the gastrojejunostomy will not interfere with the elevation of the stomach for the resection. The stomach is carefully divided with a cautery between Payr's clamps, and the margins of the division are sterilized with the cautery. The searing should be done so slowly that not a drop of blood will appear on the surface. But another and most vital purpose is achieved by this cauterization. One of the commonest dangers in operations for cancer anywhere is that of cancer implantation on the fresh tissues. The searing makes a dry charred surface which cannot sustain cancer growth. Therefore the cautery sterilizes against pygenic infections and against cancer growth and prevents bleeding—three of the most important considerations in gastric resection. As in the gastroenterostomy, protection against leakage or hemorrhage is secured by the shoemaker stitch, and to make assurance doubly sure a special invaginating silk stitch is put around each angle with a final separate layer of right-angle silk stitches. The duodenal end is inverted by a purse-string sutur.

The total number of operations on the stomach in the Lakeside surgical records are 468. Comparing previous results with those since the foregoing principles have obtained, Dr. Lower and Dr. Grile have performed, up to May, 1, 127 operations. In this series there have been two deaths.—The Therapeutic Gazette.

